# Heterotypic mouse models of canine osteosarcoma recapitulate tumor heterogeneity and biological behavior

**DOI:** 10.1242/dmm.026849

**Published:** 2016-12-01

**Authors:** Milcah C. Scott, Hirotaka Tomiyasu, John R. Garbe, Ingrid Cornax, Clarissa Amaya, M. Gerard O'Sullivan, Subbaya Subramanian, Brad A. Bryan, Jaime F. Modiano

**Affiliations:** 1 Animal Cancer Care and Research Program, University of Minnesota, St Paul, MN 55108, USA; 2 Department of Veterinary Clinical Sciences, College of Veterinary Medicine, University of Minnesota, St Paul, MN 55108, USA; 3 Masonic Cancer Center, University of Minnesota, Minneapolis, MN 55455, USA; 4 Minnesota Supercomputing Institute, University of Minnesota, Minneapolis, MN 55455, USA; 5 Department of Veterinary Population Medicine, College of Veterinary Medicine, University of Minnesota, St Paul, MN 55108, USA; 6 Department of Biomedical Sciences, Center of Emphasis in Cancer Research at the Paul Foster School of Medicine, Texas Tech University Health Sciences Center, El Paso, TX 79905, USA; 7 Department of Surgery, School of Medicine, University of Minnesota, Minneapolis, MN 55455, USA; 8 Stem Cell Institute, University of Minnesota, Minneapolis, MN 55455, USA; 9 Center for Immunology, University of Minnesota, Minneapolis, MN 55455, USA

**Keywords:** Osteosarcoma, Metastasis, Heterotypic models, Xenograft, Comparative studies, Tumor–stromal interactions

## Abstract

Osteosarcoma (OS) is a heterogeneous and rare disease with a disproportionate impact because it mainly affects children and adolescents. Lamentably, more than half of patients with OS succumb to metastatic disease. Clarification of the etiology of the disease, development of better strategies to manage progression, and methods to guide personalized treatments are among the unmet health needs for OS patients. Progress in managing the disease has been hindered by the extreme heterogeneity of OS; thus, better models that accurately recapitulate the natural heterogeneity of the disease are needed. For this study, we used cell lines derived from two spontaneous canine OS tumors with distinctly different biological behavior (OS-1 and OS-2) for heterotypic *in vivo* modeling that recapitulates the heterogeneous biology and behavior of this disease. Both cell lines demonstrated stability of the transcriptome when grown as orthotopic xenografts in athymic nude mice. Consistent with the behavior of the original tumors, OS-2 xenografts grew more rapidly at the primary site and had greater propensity to disseminate to lung and establish microscopic metastasis. Moreover, OS-2 promoted formation of a different tumor-associated stromal environment than OS-1 xenografts. OS-2-derived tumors comprised a larger percentage of the xenograft tumors than OS-1-derived tumors. In addition, a robust pro-inflammatory population dominated the stromal cell infiltrates in OS-2 xenografts, whereas a mesenchymal population with a gene signature reflecting myogenic signaling dominated those in the OS-1 xenografts. Our studies show that canine OS cell lines maintain intrinsic features of the tumors from which they were derived and recapitulate the heterogeneous biology and behavior of bone cancer in mouse models. This system provides a resource to understand essential interactions between tumor cells and the stromal environment that drive the progression and metastatic propensity of OS.

## Abstract (precis)

**Editors' choice:** We developed a system that recapitulates the heterogeneous biological behavior of bone cancer in mouse models and describe novel methods to study tumor–stromal interactions in these models.

## INTRODUCTION

Osteosarcoma (OS) is the most common malignant pediatric tumor of bone (Kansara and Thomas, 2007; Mirabello et al., 2009). Standard therapy for OS comprises neoadjuvant chemotherapy, surgery and adjuvant chemotherapy (Jaffe, 2014). The 5-year survival rates of OS patients with localized and operable OS is 60-70%, but the outcome of patients with non-resectable or metastatic OS is poor (Bielack et al., 2002; Kumta, Jan-Apr, 2016). These collective statistics belie the extreme heterogeneity of OS (Martin et al., 2012; Tan et al., 2009). Neither the histological appearance nor the propensity of the tumor cells to elaborate bone, cartilage or collagen matrices are predictive of behavior and, although recurrent molecular events have been described (Sarver et al., 2013; Scott et al., 2011; Thayanithy et al., 2012), these are yet to be adopted as prognostic or predictive biomarkers for this disease. Thus, a better understanding of the events that underlie OS tumor heterogeneity and contribute to disease progression is needed to develop effective strategies to manage OS and to improve outcomes.

OS is also the most common primary malignant tumor of bone in dogs, and it is particularly prevalent in large and giant breeds (Morello et al., 2011). In contrast to humans, OS occurs most commonly in older dogs (Fenger et al., 2014; Varshney et al., 2016). Within the extensive heterogeneity that is characteristic of both canine and human OS, important clinical and pathological features are conserved between the two species (Fenger et al., 2014; Varshney et al., 2016; Withrow et al., 1991). Adding to the weight of evidence for spontaneous OS as a homologous cellular and molecular disease of humans and dogs, we have uncovered prognostically significant gene and microRNA expression signatures that are evolutionarily conserved in human and canine OS (Sarver et al., 2013; Scott et al., 2011; Thayanithy et al., 2012).

Understanding the heterogeneous biology and behavior of OS is important to fully elucidate the pathogenesis of this disease. Most studies to date in the area of OS have been focused on studying genetic alterations. Tumor-associated stroma has remained an underrepresented area of OS research, but has been described in recent years as being complicit in the progression of other tumor types and is also an important consideration of recent anti-tumor strategies (Kang, 2016; Kawada, 2016; Sleeman, 2012; Wang et al., 2016). Robust experimental animal models that recapitulate the natural heterogeneity of OS are essential to gain insights into tumor–stromal interactions that might contribute to tumor progression, and to discover ways in which these interactions might be countered. A number of syngeneic, autochthonous and xenograft models of OS have been established in laboratory mice (Chaffee and Allen, 2013; Coomer et al., 2009; Jaroensong et al., 2012; Kanaya et al., 2011; Mohseny et al., 2012; Sampson et al., 2013; Sottnik et al., 2011; Wolfe et al., 2011), and, although many of these have examined OS pathogenesis and the effects of specific therapeutic regimens *in vivo*, few have addressed OS heterogeneity and biological behavior in the context of tumor-associated stroma and tumor–stromal interactions.

Here, we document that orthotopic canine OS xenografts preserve the biological, molecular and heterotypic biology observed in the tumors from which they were derived. Moreover, transcriptome analysis of xenograft tumors revealed a strong OS-cell-specific stromal response, which provides evidence that intrinsic genetic tumor characteristics and crosstalk between tumor and stromal cells might underlie heterogeneity of biological behavior in individuals with OS. These data provide insight into tumor–host interactions and identify targets that could play a role in treatment strategies for OS patients.

## RESULTS

### Differential growth rates at the primary site in orthotopic canine OS-1 and OS-2 xenografts

**Fig. 1.**
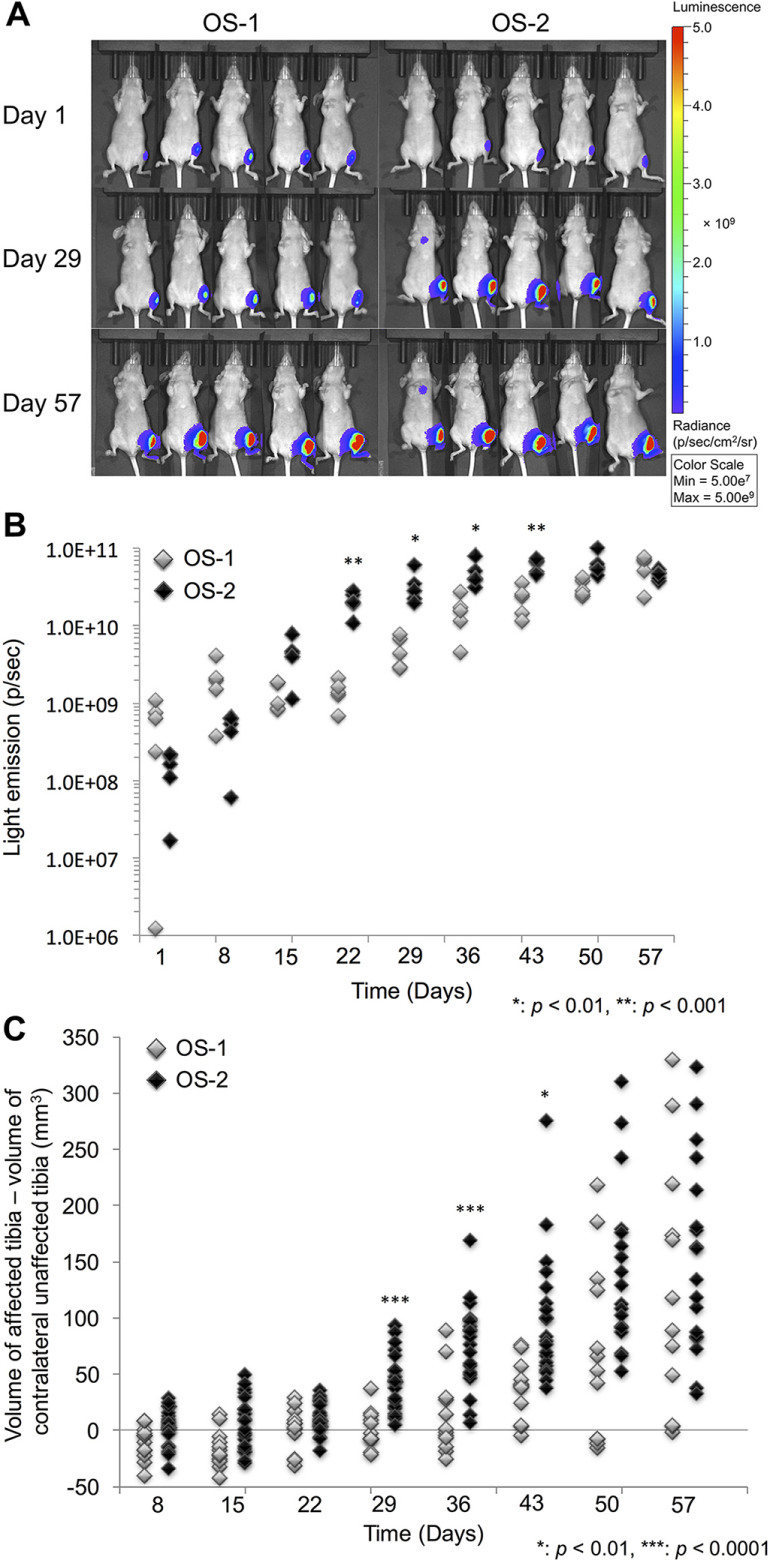
**Orthotopic canine OS-1 and OS-2 xenografts show differential growth rates at the primary site.** Athymic nude mice were injected with canine OS-1 or OS-2 cells orthotopically in the left tibia and tumor progression at the primary site was monitored by *in vivo* imaging and caliper measurements. (A) Representative examples of luciferase activity at the orthotopic site in five mice at 6 h (day 1), 4 weeks (day 29) and 8 weeks (day 57) after injection with OS-1 or OS-2 cells. Time exposures from the images for each group and from each week were different, but the radiance was adjusted to show equivalent scales in the composite. Data from the same mice that received OS-1 are shown in this figure and in Fig. 2A for day 1, but the light emission scale (in radiance=photons/sec) is adjusted in this figure to appreciate luminescence from the tumors in bone (tibiae). (B) Scatter plot showing luciferase activity for the mice in the experiment shown in panel A over time. (C) Scatter plot showing the volume of the orthotopic tumor in the left proximal tibia (minus to the volume of the unaffected, contralateral tibia) for all of the mice with orthotopic canine OS xenografts (16 mice injected with OS-1 cells and 32 mice injected with OS-2 cells) over time. Mice in B and C that received OS-1 are represented by the light symbols, and those that received OS-2 are represented by the dark symbols. The findings were analyzed with Student's *t*-test and the Holm–Sidak approach was used for multiple comparisons. Two-tailed test *P*-values are given. Significantly different growth rates between groups are denoted by *P< 0.01, **P< 0.001, ***P< 0.0001.

Development and progression of primary tumors were examined using *in vivo* imaging starting 6 h after orthotopic cell injections and then weekly for the duration of the study (Fig. 1A). Luciferase activity was detectable within 6 h in virtually all of the mice receiving OS-1 or OS-2 cells, and all of the mice showed disease progression over time. Expansion of tumor cells can be inferred from the increased luciferase emission over time; Fig. 1B shows that OS-2 intratibial xenografts had grown significantly faster than OS-1 intratibial xenografts by day 22, and this difference persisted until day 50. The results in Fig. 1C encompass a more complex process, because the physical size of the tumors in the proximal tibia would be influenced by infiltrating host stromal cells and swelling. The data confirm that OS-2 intratibial xenografts grew significantly faster than OS-1 intratibial xenografts, albeit that the effect was delayed (detectable by day 29), with this relative difference persisting until day 50 (Fig. 1B,C, Table S1). It is worth noting that neither the indirect imaging measurements nor the direct physical measurements can account for tumor invasion and loss of periosteal integrity, as is described below. Nevertheless, the data shown in Fig. 1 and Table S1 allowed us to determine that disease progression was significantly faster in animals harboring OS-2 xenografts than in animals harboring OS-1 xenografts.

### Differential metastatic propensity in orthotopic canine OS-1 and OS-2 xenografts

**Fig. 2.**
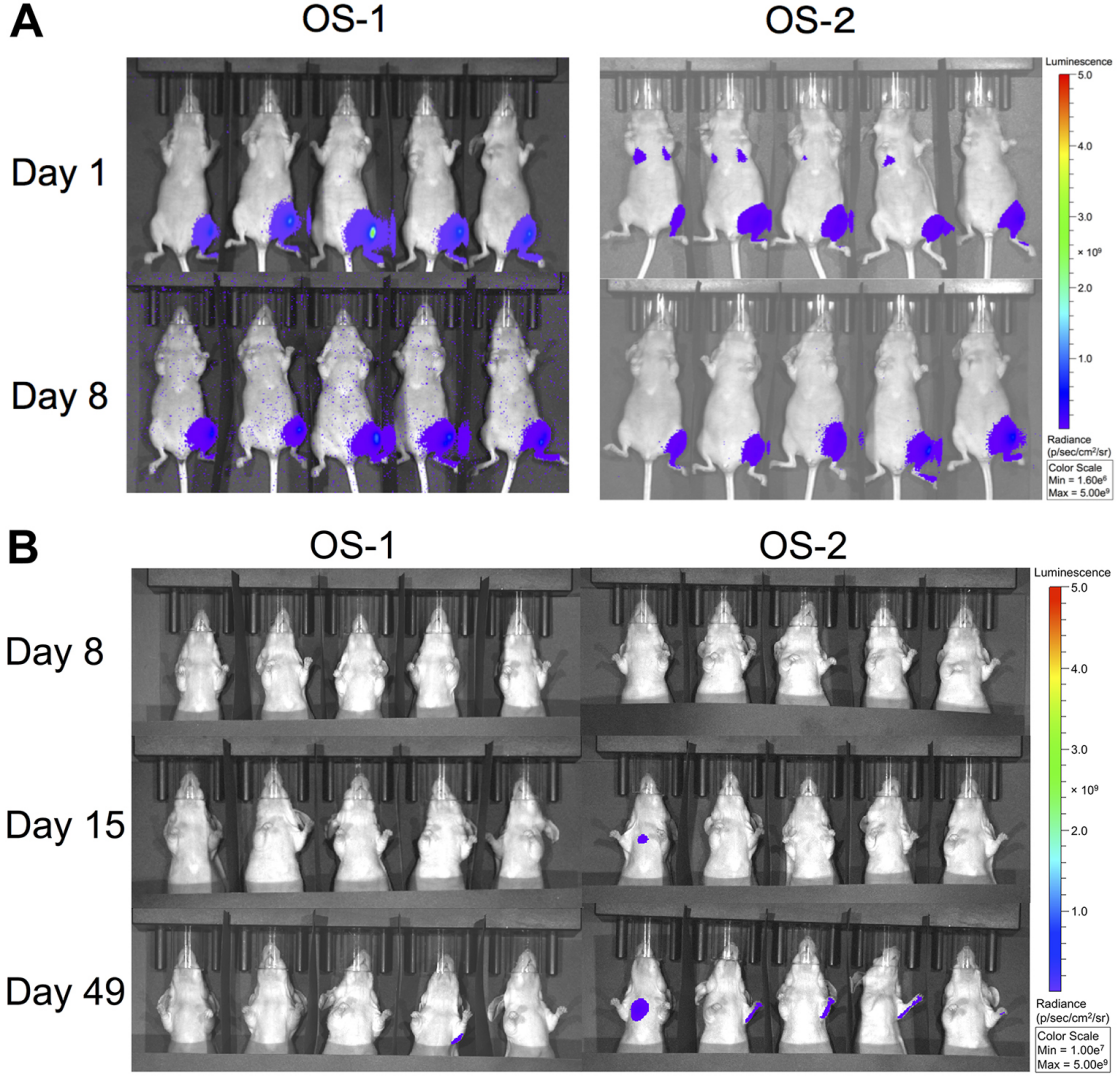
**Orthotopic canine OS-1 and OS-2 xenografts have differential metastatic propensity.** (A) Representative examples of luciferase activity at the primary site and in the lungs of five mice at both 6 h (day 1) and 8 days after intratibial injection of OS-1 and OS-2 cells. The time exposure for each image was different but, in every case, the radiance represents the same scale in the composite figure. Data from the same mice that received OS-1 are shown in Fig. 1A and in this figure for day 1, but the light emission scale (in radiance=photons/sec) is adjusted in this figure to appreciate luminescence from the tumors in lungs. A different group of mice than in Fig. 1A is shown in this figure to represent the transit of OS-2 cells to the lung. (B) Luciferase activity in the same mice shown in panel A 1 week, 2 weeks and 7 weeks after injection. The time exposure for each image was different but, in every case, the radiance represents the same scale in the composite figure. Light emission in the radii of one mouse with OS-1 and three mice with OS-2 was due to reflections from the tibial tumors; no dissemination of osteosarcoma cells was detected in the radii of any mice by histopathological examination. Data from the same mice that received OS-1 are shown in panel A and panel B for day 8, but the light emission scale in panel B is adjusted to appreciate if there was an increase in luminescence from tumors in the lungs. Different groups of mice receiving OS-2 are shown in panels A and B. The signal in the lungs at 6 h was seen consistently in mice receiving intratibial OS-2, but only one of 32 mice developed visible metastasis to the lungs by day 15. The light emission scale in panel B is adjusted to appreciate the increase in luminescence from tumors in the lungs.

We observed luciferase activity in the lungs of mice receiving intratibial OS-2 cells, but not in mice injected with OS-1 cells, within 6 h of injections (Fig. 2A). We interpreted this as evidence of systemic dissemination of OS-2 cells with accumulation in the lungs. The luciferase signal disappeared from the lungs within 1 week after tumor administration, but the presence of OS-2 cells was evident focally in the lungs of one mouse from this group again within 2 weeks after tumor administration, and the luciferase activity in this area continued to increase until the last day imaging was done for the experiment (day 49; Fig. 2B). When the mice from all the experiments were considered together, OS-2 cells achieved metastatic dissemination more rapidly than OS-1 cells (by 15, 22 and 29 days), although the rate of microscopic and macroscopic metastasis between the two groups when the experiments were terminated were not different based on imaging on day 49 (*P*=0.35) or histopathology on day 57 (*P*=0.77; Table 1).

**Table 1.**
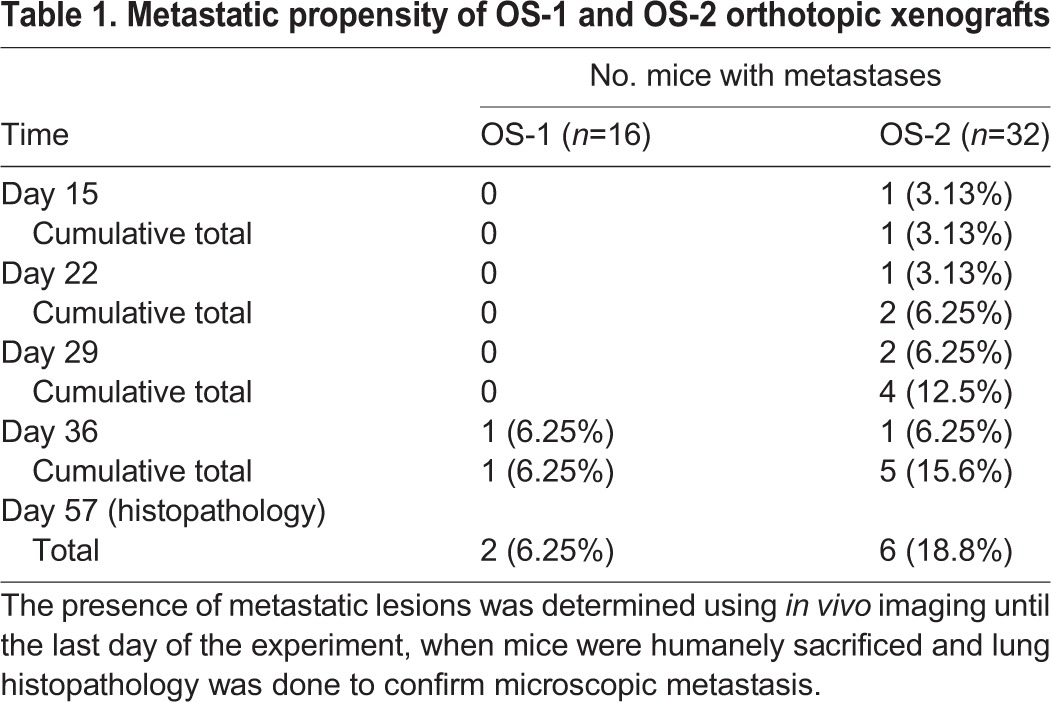
**Metastatic propensity of OS-1 and OS-2 orthotopic xenografts**

### Primary and metastatic tumors derived from orthotopic implantation of OS-1 and OS-2 cells show histological features and organization that are characteristic of canine OS

**Fig. 3.**
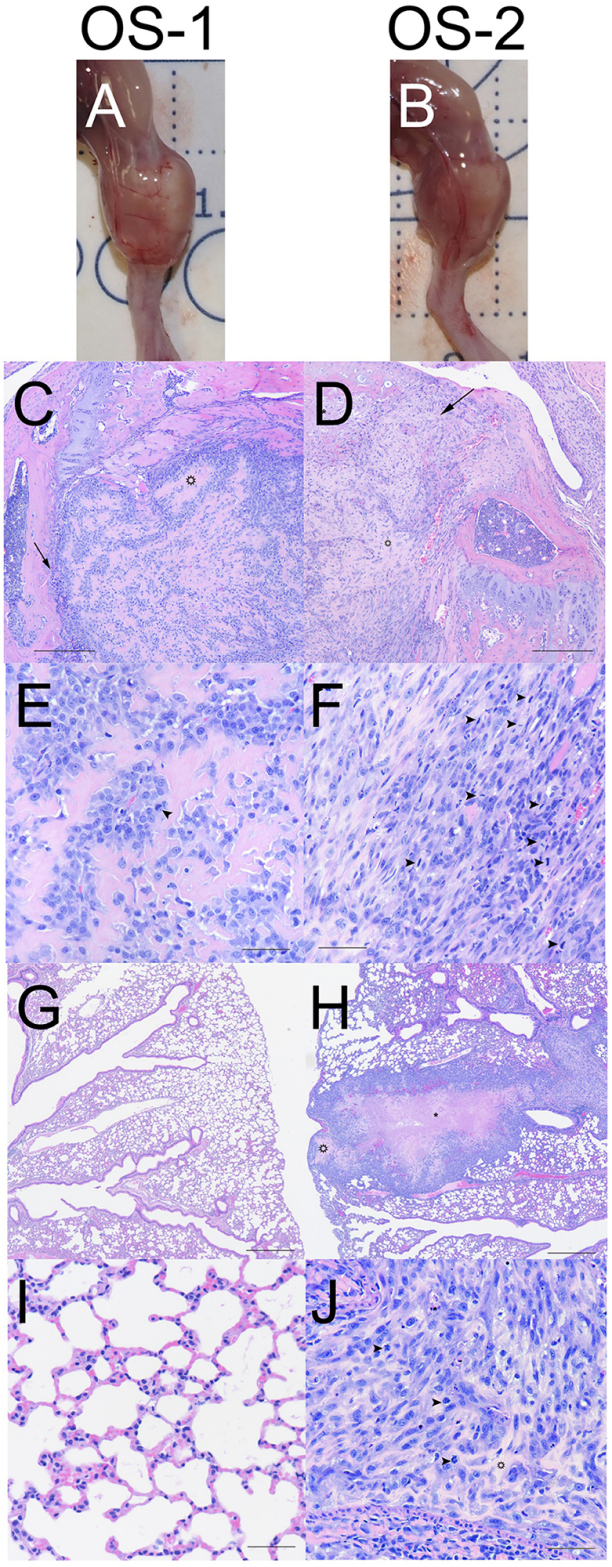
**Primary and metastatic tumors derived from orthotopic implantation of OS-1 and OS-2 cells show histological features and organization that are characteristic of canine OS.** (A,B) Images show the gross appearance of the legs from one representative mouse receiving either OS-1 (A) or OS-2 (B) cells. (C,D) Images show low-power photomicrographs of representative tumors at the primary site formed by OS-1 and OS-2 cells, respectively. The open star in C denotes an example of organized osteoid ribbons, and in D marks an example of necrosis, and arrows denote destruction of cortical bone with invasion of the epiphysis in both images. (E,F) Images show high-power photomicrographs of the tumors in C and D. Arrowheads denote mitotic figures. (G,H) Images show low-power photomicrographs of the lungs from mice receiving OS-1 (G) or OS-2 (H) cells. Areas of extensive necrosis and the pale eosinophilic matrix characteristic of OS-2 tumors are indicated by the asterisk and by the open star in H. (I,J) Images show high-power photomicrographs of the lungs from G and H. The open star denotes the pale, eosinophilic matrix characteristic of OS-2 tumors, and arrowheads in J indicate mitotic figures. Scale bars: 250 µm (C,D), 50 µm (E,F,I,J) and 500 µm (G,H).

All of the mice injected with OS-1 or OS-2 cells had evidence of gross tumor burden in the proximal tibia at necropsy on the eighth week after injection (Fig. 3A,B). Histologically, OS-1-derived tumor xenografts were characterized by relatively well-differentiated, polygonal to spindle-shaped cells that had round to oval nuclei, mild to moderate anisocytosis and anisokaryosis, and infrequent mitotic activity (Fig. 3C,E). These tumors contained organized osteoid ribbons and showed limited destruction of cortical bone and epiphyseal invasion (Fig. 3C).

In contrast, OS-2 tumors had a more aggressive appearance, with spindle-shaped, anaplastic cells that had round to elongate nuclei, moderate anisocytosis and anisokaryosis, and frequent mitotic activity (Fig. 3D,F). The cells in these tumors were embedded in a poorly organized, pale eosinophilic matrix and they showed extensive necrosis with marked destruction of cortical bone and epiphyseal invasion (Fig. 3D).

The different metastatic propensities of OS-1 and OS-2 were confirmed histologically (Table 1, Fig. 3G-J). Fewer than 20% of the mice injected with OS-2 and 7% of the mice injected with OS-1 developed metastasis by day 36 (an example of lungs without metastasis from a mouse injected orthotopically with OS-1 is illustrated by the photomicrographs shown in Fig. 3G,I). When lung metastasis was present, the histological appearance of the metastatic tumors recapitulated that of the parent tumors (Fig. 3D,F), as illustrated by the photomicrographs on one mouse receiving OS-2 orthotopically in Fig. 3H and J. In these animals, the morphology and mitotic activity of the cells and their residence in a poorly organized, pale eosinophilic matrix with extensive areas of necrosis and frequent mitotic activity were comparable to that seen in the primary tumors.

### Gene signatures of tumor cells in OS xenografts resemble those of parent cell lines

**Fig. 4.**
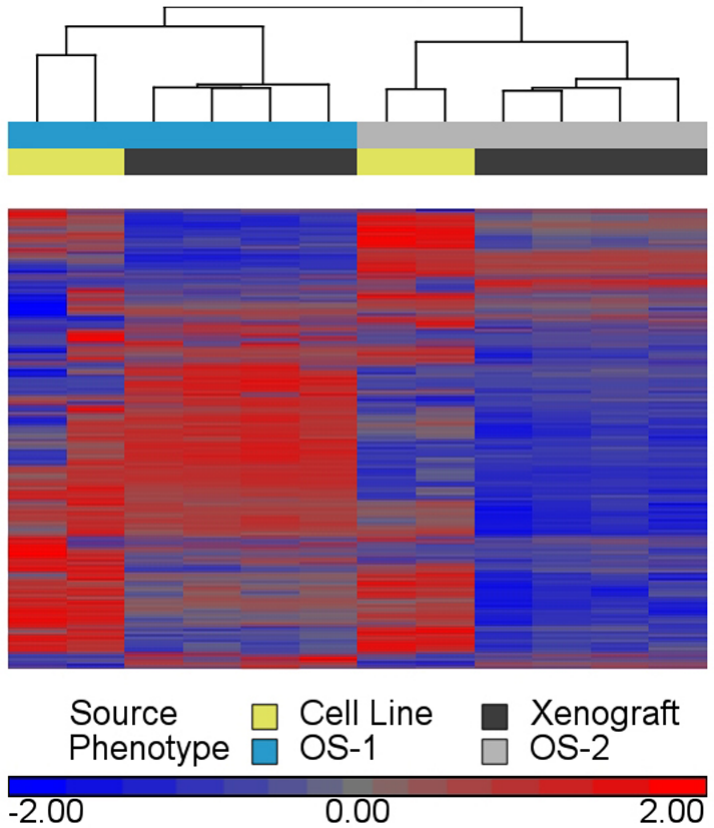
**Gene signatures of parent tumor cell lines maintained in OS-1 and OS-2 xenograft tumors.** 24,579 total canine genes were filtered to remove genes that did not have a log_2_ counts per million (CPM) mean-centered value ≥1 in at least two samples. 13,141 genes remained after filtering. The heatmap represents clustered gene-level counts with lower than mean (blue), higher than the mean (red), and mean (gray) levels of expression. Each row represents a single gene. The dendrogram represents the distance or dissimilarity between sample clusters, calculated using unsupervised hierarchical clustering on CPM values for the 13,141 filtered genes. In this dendrogram, there are two sample clusters as two branches that occur at about the same vertical distance. One of the sample clusters consists of four OS-1 (blue) xenograft tumors (black) and two parental cell line replicates (yellow), and one of these clusters consists of four OS-2 (gray) xenograft tumors (black) and two parental cell line replicates (yellow). All replicates are biological replicates.

One obstacle to using xenograft models to understand the heterogeneity of genetically complex tumors is the presumption that these tumors are unstable and will drift rapidly as they adapt to the host microenvironment. Indeed, previous data suggest that altered genomic signatures due to tumor cell plasticity and/or harsh clonal selection lead to unpredictable behavior of tumor cell lines after being transplanted into mice (Creighton et al., 2003; He et al., 2010; Hollingshead et al., 2014; Shin et al., 2013). Here, we used RNA sequencing to examine the stability of key transcriptomic properties between the parental OS cell lines and their corresponding tumor xenografts. The tumor xenografts were more similar to their corresponding parent cell lines than to each other or to the alternative cell line based on principal components analysis (data not shown) and by unsupervised clustering (Fig. 4), where tumor xenografts were assigned to the same group as their corresponding parent cell line based on the expression signatures from canine genes. When dog and mouse genes were analyzed together, expression of mouse-specific genes was not detected in the canine cell lines (Fig. S1), indicating that the mouse genes present in the tumor xenograft tissues could be accurately differentiated from the dog genes using our comparative bioinformatics approach. Furthermore, significantly larger numbers of mouse genes were detectable in OS-2 than in OS-1 xenografts, suggesting that the former tumors were more heavily infiltrated by host stroma (Fig. S1).

### OS-1 and OS-2 xenografts promote distinct tumor-associated stromal environments

**Fig. 5.**
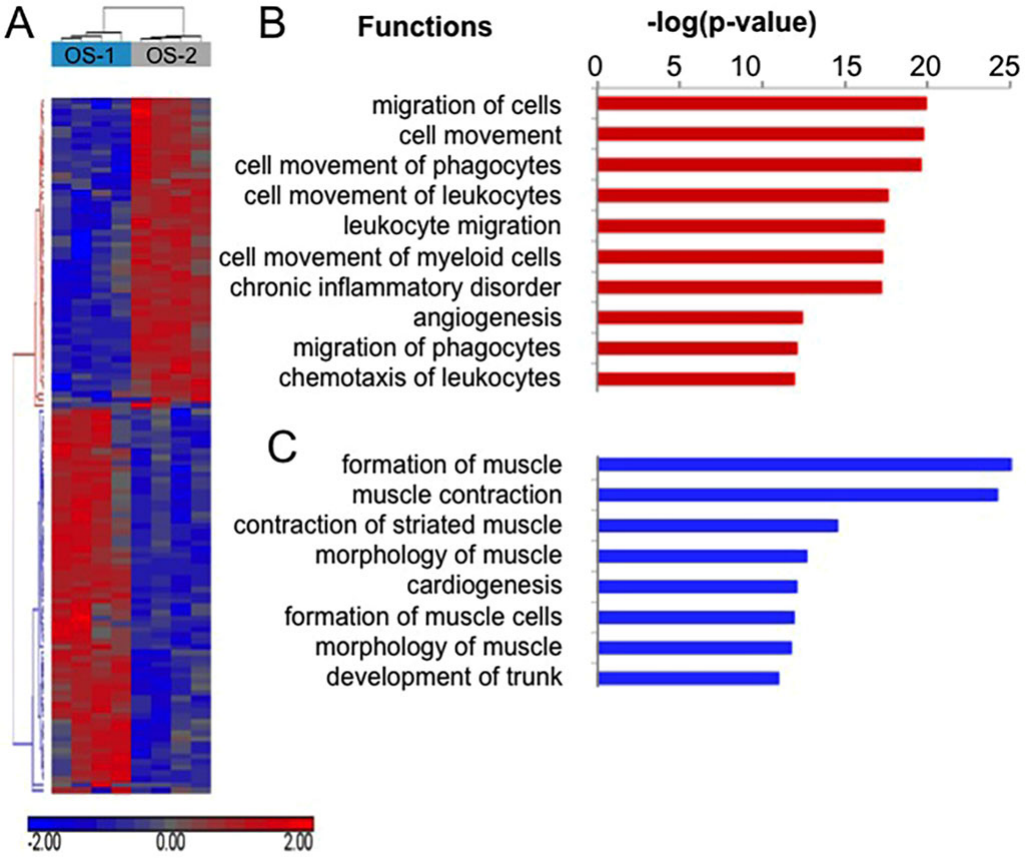
**Differentially expressed genes in OS xenografts uncover a propensity for differential stromal cell infiltrates.** EdgeR was used for pair-wise Fisher's exact test comparisons, with TMM normalization, to identify differentially expressed murine genes in tumor xenografts. Four biological replicates were used for each OS subtype. Common dispersion across all genes was calculated as 0.079 and the biological coefficient of variation (BCV) as 0.23. Mean tag-wise dispersion (individual dispersion for each gene) was calculated as 0.095. Using statistical significance criteria of FDR-adjusted *P*<0.005 and log_2_ fold change >2, 482 differentially expressed murine genes were identified. After identifying differentially expressed genes (DEGs), log-transformed and mean-centered counts per million (CPM) values for 47,997 canine and murine genes were generated. The Pearson distance similarity metric and average linkage clustering method was used for hierarchical clustering of log_2_ CPM values for the 482 differentially expressed murine genes. See Table S1 for detailed gene lists. (A) Heatmap shows clustered gene-level counts with lower than mean (blue), higher than the mean (red) and mean (gray) levels of expression. Each row represents a single gene. The dendrogram of the horizontal axis of the heatmap shows two sample clusters; OS-1 (blue) and OS-2 (gray) xenografts are in separate sample groups. The rows of the heatmap (vertical axis) cluster into two highly correlated groups. Rows colored in red in the vertical dendrogram are murine genes that are upregulated in OS-2 xenografts, whereas rows colored in blue are downregulated relative to OS-1 xenografts. (B,C) Enriched pathway and functional classification analyses of DEGs were performed using IPA according to row cluster designation. (B) Upregulated genes, red; (C) downregulated genes, blue.

To determine the nature of the stromal interactions and the identity of the infiltrating cells in the xenografts, we performed pairwise Fisher's exact test comparisons, with trimmed mean of *M*-values (TMM) normalization of gene counts, to identify the differentially expressed murine genes in tumors from each group (OS-1 and OS-2). Using a false discovery rate (FDR)-adjusted *P*-value of <0.005 and log_2_ fold change >2, we identified 482 genes that were expressed at significantly different levels between the two groups (Fig. 5A; Table S2). Pathway analysis of these 482 differentially expressed murine genes was done by MetaCore software. The top ten most enriched pathways suggest immune and inflammatory themes that modulate IL-17, TGF-β signaling, the complement system, and patterning behavior and cytoskeletal remodeling with involvement of Rho GTPases (Table 2).

**Table 2.**
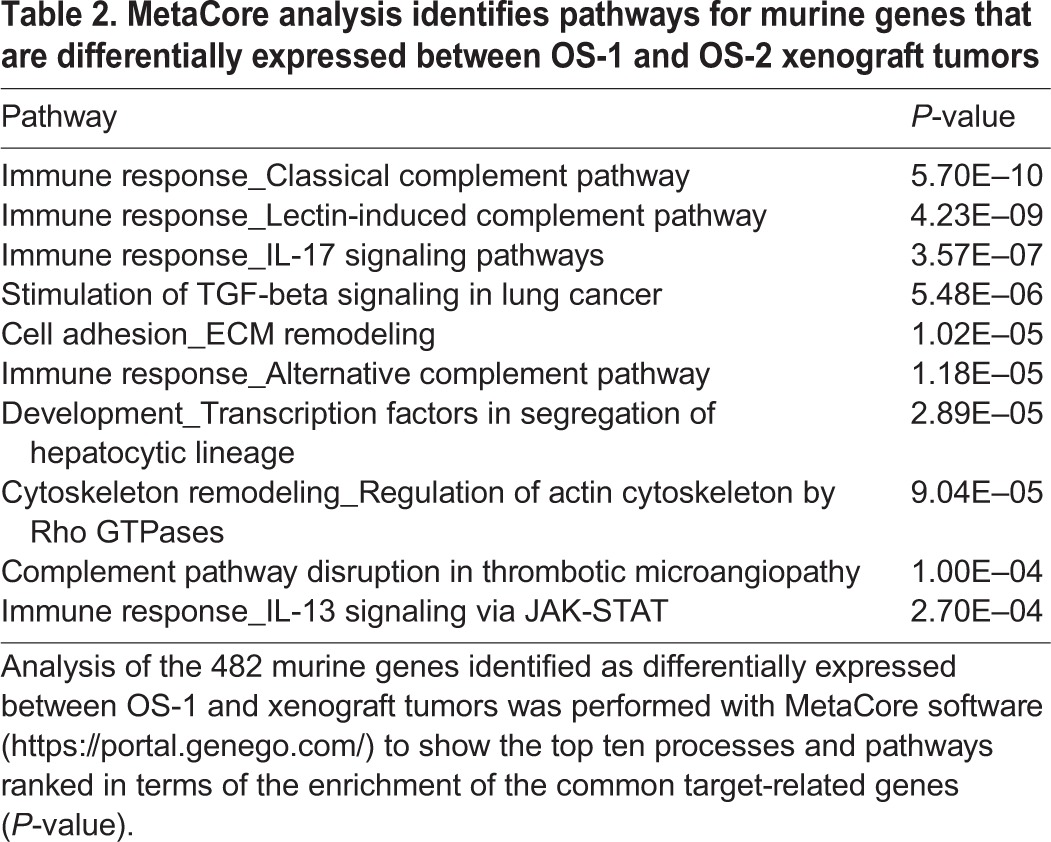
**MetaCore analysis identifies pathways for murine genes that are differentially expressed between OS-1 and OS-2 xenograft tumors**

We looked at upstream regulators of these 482 differentially expressed murine genes by Ingenuity Pathway Analysis (IPA). The most significant, predicted activated upstream regulators in OS-2 (worse prognosis), relative to OS-1 tumor xenografts, were *CEBPB* and *NFKB1* (*P*-value 5.54E–10 and 3.94E–09, respectively), whereas the most significant predicted inhibited upstream regulator was *MEF2C* (*P*-value 2.54E–23) (Table S3). The retinoblastoma tumor suppressor gene (*RB1*) was also among the predicted significant upstream regulators (*P*-value 1.25E–04) showing inactivation in OS-2 xenograft tumors, as we would have predicted based on our previous work (Scott et al., 2015) (Table S3).

To better understand the unique differences between OS-1 and OS-2, we considered the upregulated and downregulated murine genes in OS-2 as separate lists and used IPA to identify enriched biological functions and transcription factors that regulate these genes. The 482 differentially expressed murine genes included 240 that were upregulated (Fig. 5B) and 242 that were downregulated (Fig. 5C) in OS-2 tumor xenografts relative to OS-1 tumor xenografts. The most upregulated murine gene in the OS-2 xenografts was *Mcpt1* (+11.25 fold), whereas the most downregulated murine gene was *Nkx2-1* (–10.97 fold) (Table S2).

Based on biological function and processes, the most upregulated murine genes in OS-2 tumors were proteases, metallopeptidases, cytokines and chemokines involved in cell movement, leukocyte migration, inflammation and angiogenesis (Fig. 5C, Table S2). By contrast, the most downregulated genes in OS-2 tumor xenografts were transcriptional regulators of cellular differentiation and cell cycle involved in the formation and morphology of muscle (Fig. 5C, Table S2).

Upstream regulators predicted to modulate expression and activity of the 240 upregulated expressed murine genes in the OS-2 tumor xenografts included the T-helper cell type-17 (Th17)-activating cytokines TGF-β (*P*-value 1.26E–27), IL-1β (*P*-value 9.07E–25) and IL-6 (*P*-value 9.03E–22) (Table S4).

The top upstream regulators predicted to modulate expression and activity of the 244 downregulated murine genes in the OS-2 xenografts were *MEF2C* and *MYOD1* (*P*-value 1.15E–24 and 2.16E–15, respectively) (Table S5). *MEF2C* and *MYOD1*, both predicted as being inhibited in OS-2 xenografts and activated in OS-1 tumors, are important in promoting transcription of muscle-specific target genes and play a role in muscle differentiation.

## DISCUSSION

In this study, we established a novel approach using mouse xenografts to study the heterogeneity and biological behavior of OS *in vivo*. Specifically, this approach creates opportunities to examine tumor-intrinsic properties, as well as organotypic tumor–stromal interactions that influence tumor progression.

We injected cells at the orthotopic site to simulate the biology of the spontaneous disease. A comprehensive review by Talmadge et al., (2007) described the advantages of orthotopic xenografts over subcutaneous xenografts for solid tumors. Importantly, the anatomical site of implantation needs to be considered carefully because the biological behavior of tumors is dependent on the intrinsic properties of both tumor cells and host factors (which differ between tissues and organs). The microenvironment in subcutaneous xenografts consists of desmoplastic mouse stromal cells that do not resemble the organization seen in autochthonous tumors (Delitto et al., 2015). These properties also apply to OS: Rosol and colleagues showed that orthotopic canine OS xenografts in nude mice produced osteoid matrix and metastasized spontaneously, whereas subcutaneous xenografts did not (Wolfe et al., 2011).

The applicability of cell lines to understand tumor heterogeneity has similarly been challenged (Choi et al., 2014; McIntyre et al., 2015). However, our data show that heterogeneity of biological behavior (including metastatic propensity) can be recapitulated to a limited extent in tumors from cell lines, but more readily by utilizing multiple cell lines that cover the spectrum of tumor behavior. Another impediment that has been articulated is genetic drift, where cell lines that adapt to grow in culture no longer resemble the genetic makeup of the parental tumors (Daniel et al., 2009). Yet, our data show that the major genetic drivers that distinguish the two canine OS cell lines *in vitro* were retained in the orthotopic xenografts. In addition to stability of the transcriptome, the cell lines show stable morphology from the primary canine tumors to the primary orthotopic tumors (Scott et al., 2015), and to the metastatic tumors. Confirmation of this remarkable genetic and morphologic stability over many passages was essential to validate the utility of our model to understand OS tumor heterogeneity.

As predicted from the original behavior of the spontaneous tumors in the dogs and from their gene and microRNA expression signatures (Sarver et al., 2013; Scott et al., 2011; Thayanithy et al., 2012), the logarithmic expansion phase of OS-2 primary xenografts was faster than that of OS-1 primary xenografts. However, both cell lines seemed to reach the tumor endpoints at approximately the same time. We believe that two factors might account for this. First, the tumors are growing within a cavity surrounded by bone and, despite the fact that OS-2 xenografts showed greater epiphyseal destruction and invasion, the bone constrains the maximum size achievable by the primary tumors within the experimental time frame. Second, mice with OS-2 xenografts did not show greater morbidity than mice with OS-1 xenografts, determined by the absence of lameness, ambulatory deficits and other behaviors associated with chronic pain. This could be due to adaptive behavior of prey species to hide pain (Arras et al., 2007); however, previous work has shown unequivocally that painful intramedullary bone tumors produce behavioral changes in mice (Pacharinsak and Beitz, 2008). We should note that these cell lines accurately represent the biological behavior of the tumors from which they were originally derived, and more broadly the classification of more aggressive and less aggressive tumors (Moriarity et al., 2015; Sarver et al., 2013; Scott et al., 2011, 2015; Thayanithy et al., 2012). Furthermore, such properties have been verified independently by other groups using one of these cell lines (Wolfe et al., 2011), and they generally extend to human and murine osteosarcoma (Moriarity et al., 2015) (M.C.S., unpublished). Still, data from two cell lines should be interpreted with caution because osteosarcomas are extremely heterogeneous, both genetically and biologically (Varshney et al., 2016). It will thus be important to document the fidelity with which this model is able to recapitulate such heterogeneity.

Beyond growth at the primary site, biological behavior can be quantified by metastatic propensity and successful spread to distant sites. Again, the predictions from the original spontaneous tumors were confirmed experimentally in our models. OS-2 cells were a representative example from a group of highly aggressive tumors (worse prognosis) that showed high expression of cell-cycle- and DNA-damage-repair-associated genes, with concomitant reduced expression of a complement of genes that defined ‘microenvironment interactions’ (Scott et al., 2011). This reduced expression of molecules that mediate local cell communication could explain, at least in part, the observation that cells injected intratibially achieved rapid systemic distribution, spreading to the lungs within 6 h; i.e. there was nothing to hold the cells in place, and they had no preference to remain in the local bone environment.

We cannot completely exclude the possibility that metastasis is driven by selection of cells that acquire (or previously harbored) mutations or epigenetic events that favor dissemination to and survival in the lung, and, in fact, both mechanisms (niche conditioning and selection) could be operative in this model. If this were the case, however, and given the reproducibility of results over multiple experiments and the low probability that cells would acquire the same stochastic mutations repeatedly, one would have to argue that such mutations or epigenetic changes pre-exist in a subset of cells within these cell lines.

A previous study (Garimella et al., 2013) used *in vivo* imaging to show tumor cells in the lungs within 2 weeks after orthotopic implantation of OS cells; however, we are not aware of any previous studies showing dissemination of OS cells into the lungs on the day of implantation. In our experiments, the luciferase signals disappeared from the lungs in all of the mice receiving OS-2 xenografts within 24 h, and they were only visible again in one of the 32 mice within 2 weeks and eventually in six of 32 mice that received OS-2 cells by the end of the experiment. This suggests that the lung niche required prior conditioning by OS-2 tumors in order to become receptive for metastatic colonization. Furthermore, our results suggest that, even though both OS-1 and OS-2 cell lines can establish a metastatic niche, they do so with different kinetics, creating a suitable model to study intrinsic differences in metastatic propensity, as well as host-related factors that contribute to the metastatic niche in OS.

Based on these observations, we could propose two distinct mechanisms for the different metastatic potential of OS-1 and OS-2 xenografts. One noted above is that OS-2 cells might have greater metastatic potential due to their interaction with the local microenvironment in the bone, which leads to reduced retention, and potentially to an increased capability to condition the distant site. The alternative possibility is that, as shown in Fig. 2, OS-2 cells seed the lungs shortly after inoculation and, even though many of these cells might leave the lungs or die, accounting for the loss of luciferase signal by 24 h, some cells remain and eventually form the pulmonary lesions (i.e. equivalent to seeding or colonization by intravenous inoculation). We favor the first possibility because preliminary experiments suggest that OS-1 and OS-2 cells have low efficiency of pulmonary colonization upon intravenous injection. Nevertheless, additional experiments will be necessary to formally exclude the second possibility.

Our results differ from those of Rosol's group (Wolfe et al., 2011), which showed development of multiple lung metastases in all of the mice that received orthotopic injections with canine OSCA-40 cells (OS-2). This is almost certainly due to the fact that we terminated our experiments after 8 weeks, whereas Rosol et al. continued their experiments for up to 12 weeks. We thus surmise that OS-2 and other aggressive OS cell lines can be used to investigate therapeutic interventions to delay or prevent OS metastasis in the minimal residual disease setting, whether attained through amputation or through administration of cytoreductive chemotherapy. Future experiments could further investigate the intrinsic biology of the tumors and mechanisms of drug resistance, as well as preclinical interventions to delay or prevent metastatic dissemination, especially by modeling the current standards of care, which combine surgery with neoadjuvant and adjuvant chemotherapy, and particularly expanding the model to leverage multiple available human and canine OS cell lines (Lauvrak et al., 2013; Mohseny et al., 2011; Scott et al., 2011).

Finally, genomic stability might be a peculiar feature of OS xenografts. Consistent with our previous study (Scott et al., 2015), our present results indicate that functional *RB* can be stably maintained in OS cells, and that its loss in the tumor cell compartment is associated with a more aggressive phenotype of rapid growth and increased metastatic propensity. In addition, other studies have shown that gene expression patterns and copy number alterations were preserved in patient-derived OS cell lines and xenograft tumors (Kuijjer et al., 2011; Mayordomo et al., 2010). Yet, we are not aware of any previous studies describing the relationship between intrinsic gene signatures of OS tumor cells with distinctly different biological behaviors and host stromal cells.

Highly expressed mouse genes present in the OS-2 xenografts were associated with B-cell signaling, inflammation and immune response, whereas mouse genes in the OS-1 cells xenografts were associated with patterning, and especially with muscle formation. Increased expression of myogenic regulators in mouse stromal cells in OS-1 xenografts raises interesting questions regarding possible effects of OS-1 tumor cells on marrow-derived mesenchymal stromal cells. Interestingly, myogenic regulators have been implicated in human oncogenesis. For instance, expression of *MYOD1* can predict patient survival in lung cancer patients (Jiang et al., 2015) and high expression of *MEF2C* is associated with poor outcome in acute myeloid leukemia (AML) patients (Laszlo et al., 2015). Moreover, myogenic transcription factors have been shown to regulate metastasis in soft-tissue sarcomas, suggesting that further investigation of these factors be done in other sarcomas, including OS (Dodd et al., 2016). Importantly, *MEF2C* was recently identified as a candidate tumor suppressor gene in OS in a forward genetic screen (Moriarity et al., 2015).

Intriguingly, the most downregulated murine gene in the OS-2 xenografts was the transcription factor *Nkx2-1*, which is known to regulate lung epithelial cell morphogenesis and differentiation. Downregulation of *NKX2-1* has been shown to precede dissemination of lung adenocarcinoma cells (Caswell et al., 2014). *NKX2-1* amplification has been reported in one human OS patient but there are no reports of downregulation or loss of *NKX2-1* in OS patients (Egas-Bejar et al., 2014).

Activated TGF-β, IL-6 and IL-1β were the most significant upstream regulators of highly expressed genes from stromal cells in the OS-2 xenografts. This is especially intriguing because this would normally be associated with a pro-inflammatory Th17 response. Such a response cannot happen in athymic nude mice, which lack T cells; in fact, it is important to recognize that a limitation of this model is the fact that the full complement of the T-cell immune response cannot be studied in the immunocompromised mouse strains that provide receptive hosts for tumor xenotransplantation. This then creates a gap that will have to be addressed using syngeneic or autochthonous, immunocompetent animal models.

In conclusion, we have developed xenograft models that recapitulated the heterogeneous biological behavior of OS. These models will be useful to understand the mechanisms that drive progression and metastasis of OS because they are expandable into additional cell lines to represent a wider spectrum of disease.

## MATERIALS AND METHODS

### Cells and culture conditions

Two canine OS cell lines representing previously described ‘less aggressive’ and ‘highly aggressive’ molecular phenotypes (OS-1 and OS-2, respectively), were used in this study (Scott et al., 2011, 2015). OS-1 and OS-2 are derivatives of the OSCA-32 and OSCA-40 cell lines (Scott et al., 2011, 2015). Specifically, OS-1 represents a subline that successfully established tumors after orthotopic implantation, because the parental OSCA-32 did not establish heterotopic or orthotopic tumors on every occasion. OS-2 represents the parental OSCA-40, which reliably formed tumors after orthotopic implantation in every experiment done (Scott et al., 2015; Wolfe et al., 2011).

Cell lines were validated using short tandem repeat (STR) profiles by DNA Diagnostics Center (DDC Medical) (Fairfield, OH). OS-1 and OS-2 cells were modified to stably express green fluorescent protein (GFP) and firefly luciferase as described (Scott et al., 2015) and used for orthotopic injections in mice. After transfection and selection, we confirmed that the GFP/luciferase construct was stably integrated in each cell line by fluorescence *in situ* hybridization, and we corroborated that the two cell lines had approximately equivalent luciferase activity on a per cell basis using conventional luciferase assays (Scott et al., 2015). All cell lines were grown in DMEM (Gibco, Grand Island, NY) containing 5% glucose and L-glutamine, supplemented with 10% fetal bovine serum (Atlas Biologicals, Fort Collins, CO), 10 mM 4-(2-hydroxyethyl)-1-piperazine ethanesulphonic acid buffer (HEPES) and 0.1% Primocin (InvivoGen, San Diego, CA), and cultured at 37°C in a humidified atmosphere of 5% CO_2_. Canine OS cell lines are available for distribution through Kerafast, Inc. (Boston, MA). Each cell line was passaged more than 15 times before the experiments when they were inoculated into mice.

### Mice

Six-week-old female, athymic nude mice (strain NCr^nu/nu^) were obtained from Charles River Laboratories (Wilmington, MA). The University of Minnesota Institutional Animal Care and Use Committee approved protocols for mouse experiments of this study (protocol no.: 1307-30806A).

### Tumor xenografts

Eight animals per group provide >95% power to identify a 15% change in the median time to tumor when the σ for both populations is <2.0 and the acceptable α error is 5% (*P*<0.05). Experimental replicates increased statistical robustness, accounting for the expected heterogeneity.

Four replicate experiments were done to assess orthotopic growth and metastatic dissemination of OS-1 and OS-2 cells. For the first pilot experiment, groups of three mice were used to validate the approach. All of the mice receiving OS-1 xenografts showed successful implantation, but only two of the three mice receiving OS-2 xenografts showed successful implantation. For the second experiment, groups of 16 mice were used to establish significance. In this experiment, all of the mice receiving OS-2 xenografts showed successful implantation, but eight mice injected with OS-1 xenografts had significant adverse effects during anesthesia and were not recovered (i.e. they were humanely euthanatized). For the third experiment, we inoculated nine mice with OS-2 cells to verify the unexpected effects of rapid dissemination to the lung. No mice received OS-1 for this experiment. Finally, for the fourth experiment, we inoculated five mice with each cell line (OS-1 or OS-2) to achieve a biological replicate of experiment two, maintaining the sample size at a number to maximize a positive outcome. Appropriate censoring was used to include all animals in the analyses, only excluding any which succumbed acutely or subacutely during the intratibial injection procedure. Thus, 16 mice inoculated with OS-1 were included in the analyses of tumor growth, and 32 mice inoculated with OS-2 were included in the analyses of tumor growth.

We previously determined that four samples per group approximate the point of minimal returns using large genomic datasets for gene expression profiling (Tamburini et al., 2009), and these estimates hold true from microarrays to RNAseq where the fidelity of replication within samples is high, despite orders of magnitude more data (see analysis of RNA sequencing below).

Animals were assigned to separate cages (four animals each) in random order for each experiment. All of the animals in each cage received the same treatment. OS-1 and OS-2 cells expressing GFP and firefly luciferase were injected intratibially. Mice were anesthetized with xylazine [10 mg/kg body weight, intraperitoneally (IP)] and ketamine (100 mg/kg, IP), and 1×10^5^ cells suspended in 10 µl of sterile PBS were injected into the left tibia using a tuberculin syringe with 29-gauge needle. Buprenorphine (0.075 mg/kg, IP  every 8 h; Buprenex^®^, Reckitt Benckiser Healthcare, Richmond, VA) was used for pain control over the first 24 h after injection of tumor cells, and prophylactic ibuprofen administrated in the water was used over the next 3 days.

Tumor growth was monitored by measuring width (W) and length (L) of the proximal tibia and the stifle joint weekly using calipers, as well as by *in vivo* imaging as described (Kim et al., 2014). Bioluminescence imaging (Xenogen IVIS spectrum, Caliper Life Sciences, Hopkinton, MA) was done after injection of D-luciferin (Gold Biotechnology, St Louis, MO) following isoflurane inhalant anesthesia and analyzed with Living Image Software (Caliper Life Sciences). Bone tissue volume (V) was calculated from both tibiae using the equation V=L×W^2^×0.52 (Banerjee et al., 2013) and tumor volume was estimated by subtracting the normal bone tissue volume of the contralateral unaffected (right) tibia from the volume of the affected (left) tibia.

Mice were observed for up to 8 weeks or until tumor endpoint criteria were reached (ill thrift, tumor reaching 1 cm in the largest diameter, visible lameness, pain or severe weight loss), at which time they were humanely euthanized with pentobarbital sodium and sodium phenytoin solution (Beuthanasia-D Special^®^, Schering-Plough Animal Health, Union, NJ). Primary bone tumors and lung tissues were dissected and a portion of each was stored at −80°C for RNA extraction. The remaining tissues were fixed in 10% neutral-buffered formalin, and processed for routine histological examination.

Luciferase activity and tumor sizes were compared using multiple *t*-test and Holm–Sidak method with Prism 6 software (GraphPad). *P*<0.05 was used as the level of significance.

### RNA extraction, library preparation and RNA sequencing

Total RNA was extracted from primary intratibial tumors and from cell lines using the miRNeasy Mini Kit (QIAGEN, Valencia, CA). RNA integrity was examined using Agilent 2100 Bioanalyzer (Agilent Technologies, Santa Clara, CA) and RNA integrity number (RIN) values of all samples were >8.0. Sequencing libraries were prepared with the TruSeq Library Preparation Kit (Illumina, San Diego, CA). RNA sequencing (100-bp paired-end) with HiSeq 2500 (Illumina) was done at the University of Minnesota Genomics Center (UMGC). A minimum of ten-million read-pairs was generated for each sample.

### Analysis of RNA sequencing data

Initial quality control analysis of RNA sequencing (FASTQ) data for each sample was performed using the FastQC software (version 0.11.2; http://www.bioinformatics.babraham.ac.uk/projects/fastqc). FASTQ data were trimmed with Trimmomatic (Bolger et al., 2014). HISAT2 (Kim et al., 2015) was used to map paired-end reads from eight xenograft tumors (four tumors of OS-1 and four tumors of OS-2) and four parental cell-line samples (two each for OS-1 and OS-2 cell lines). For accurate alignment of sequencing reads to canine and murine genes within xenograft tumors, a HISAT2 index for mapping was built from a multi-sequence fasta file containing both the canine (canFam3) and murine (mm10) genomes. Insertion-size metrics were calculated for each sample using Picard software (version 1.126) (http://picard.sourceforge.net). Samtools (version 1.0_BCFTools_HTSLib) was used to sort and index the bam files (Li et al., 2009). Transcript abundance estimates were generated using the Rsubread featureCounts program for differential gene expression analysis (Liao et al., 2014).

Gene counts for each xenograft sample were imported into RStudio (v. 3.2.3) (http://www.rstudio.com) for differential gene expression (DGE) analysis with EdgeR (Robinson et al., 2010; Zhou et al., 2014). Lowly expressed genes were removed by filtering. A gene was considered expressed if it had log_2_-transformed read counts per million (CPM) >1 in at least two of the eight xenograft tumors. Biological variation within xenograft sample groups was estimated by common dispersion and biological coefficient of variation (BCV) calculations (Robinson et al., 2010). Pairwise empirical analysis of differential gene expression was performed on sample groups (OS-1 and OS-2) using Fisher's exact test for two-group comparisons with TMM normalization (Robinson and Oshlack, 2010). Tagwise dispersion (individual dispersion for each gene) was used to adjust for abundance differences across biological replicates (*n*=4) within each xenograft group (OS-1 and OS-2). Gene counts as CPM were imported into Partek Genomic Suite for clustering analysis and visualization. The Pearson similarity metric and average linkage clustering method were used for hierarchical clustering of mean-centered CPM values. Enriched pathway and functional classification analyses of DGEs were performed using QIAGEN's Ingenuity^®^ Pathway Analysis (IPA^®^, QIAGEN Redwood City, www.qiagen.com/ingenuity). The reference set for all IPA analyses was the Ingenuity Knowledge Base (genes only) and human Entrez gene names were used as the output format. To understand the high-level functions and utilities that each gene identified as differentially expressed between OS-1 and OS-2 was associated with, we utilized MetaCore software (Thompson Reuters) to identify statistically over-represented cellular processes in the dataset.
